# The signaling cascade of induction and maintenance of ES cell diapause

**DOI:** 10.21203/rs.3.rs-4946357/v1

**Published:** 2024-09-03

**Authors:** Alexander Tarakhovsky, Tuo Zhang, Ryan Marina, Sarah Veugelen, Pal Mander, Rabinder Prinjha, Anne Schaefer, Karen Adelman

**Affiliations:** Laboratory of Immune Cell Epigenetics and Signaling, The Rockefeller University; The Rockefeller University; Harvard Medical School; Max Planck Institute for Biology of Ageing; GlaxoSmithKline; Glaxo SmithKline; Harvard Medical School

## Abstract

Nutrient deficiency during pregnancy in numerous animal species can induce the state of embryonic diapause. Diapause is characterized by changes in protein and gene expression that minimize the organism’s reliance on external energy sources and ensure survival. Remarkably, the systematic changes associated with diapause appear to spare the gene expression program that supports embryonic cells’ maintenance in the pluripotent state. The phenomenon of the differentiation “freeze” during diapause can be reproduced *in vitro*. Mimicking nutrient deficiency by pharmacological inhibition of mTOR induces the diapause-like state in ES cells without affecting ES cell pluripotency. We discovered a connection between mTOR signaling and the chromatin-bound bromodomain and extra-terminal (BET) transcriptional regulator BRD4, showing a key role of BET-protein in the induction of diapause-like state in ES cells. mTOR inhibition rapidly and negatively impacts BRD4 binding to chromatin, which is associated with changes in gene expression that can contribute to diapause. Conversely, pharmacological inhibition of BET-protein circumvents the diapause dependence on mTOR inhibition and causes the diapause-like state. BET-repressed diapause-like ES cells retain the undifferentiated pluripotent state, which is associated with upregulation of a functionally linked group of genes encoding negative regulators of MAP kinase (MAPK) signaling and inactivation of MAP kinase. The transcriptional switch-off of MAP kinase following chronic BET inhibition imitates the transcriptional de-repression of MAP kinase negative regulators in response to mTOR inhibition. Mechanistically, suppression of mTOR or BET-protein leads to a profound decline in Capicua transcriptional repressor (CIC) at promoters of key negative regulators of MAP kinase. The discovered mTOR-BRD4 axis in the induction of diapause and the rapid transcriptional shut-off of differentiation program is likely to play a major role in the maintenance of embryonic diapause *in vivo*, as well as in controlling of the undifferentiated state of various types of stem cells during diapause-like metabolic dormancy.

## Introduction

Embryonic diapause is a distinct organism phenotypic state that occurs in response to a limited nutrient supply to newly conceived embryos in numerous species of animals, including mice^1^. Diapause is characterized by reduced anabolism and diminished levels of various biosynthetic processes, including protein synthesis and gene expression.^2–5^ Remarkably, diapause-associated translational and transcriptional changes have no evident impact on the differentiation potential of the embryos that, upon restoration of the nutrient supply, resume normal growth and give rise to healthy progeny.^1,6^ The mechanism by which diapause embryos can maintain the undifferentiated potential for extended periods is unknown.

Diapause *in vivo* can be phenotypically imitated *in vitro* by nutrient deprivation of *ex vivo* isolated mouse blastocysts^3^ or cultured embryonic stem (ES) cells^3,4^. The eukaryotic cell response to nutrient supply is governed by the mTORC1/2 protein complexes^7^. Accordingly, pharmacological suppression of mTOR imitates nutrient deprivation and causes a diapause-like state *in vitro*^3,4^. This diapause-inducing effect of mTOR inhibition has been mostly linked to translational changes^3,8^. We identified a novel link between the activity of mTOR and gene transcription via BRD4, a member of the bromodomain and extra-terminal (BET) protein family that plays a key role in gene regulation^9,10^. The mTOR-BRD4 axis acts as reinforcer of the negative effect of nutrient deprivation on gene transcription. Conversely, long-term suppression of BET circumvents diapause dependence on mTOR and induces the diapause-like state in ES cells.

## Results

### mTOR inhibition has a rapid and strong negative effect on BRD4 subnuclear organization and chromatin binding

Treatment of ES cells with Torin1, which inhibits both mTORC1 and mTORC2 (hereafter mentioned as mTOR), alters the BRD4 organization into the supramolecular nuclear domains (BRD4 + speckles) and BRD4 association with specific gene targets (Fig. 1). Treatment with Torin1 results in a rapid reduction in the BRD4-containing nuclear speckle numbers (Fig. 1a) without affecting the overall BRD4 expression levels (Extended Data Fig. 1a). The Torin1 effect on BRD4 speckles is within the range of the effect of the pan-bromodomain inhibitor I-BET151 (Fig. 1b). The I-BET affects the bromodomain-mediated BET binding to the acetylated histone H4 and, to minor degree, to other acetylated nuclear proteins^11^. The mTOR inhibition has no effect on histone H4 acetylation genome-wide (Extended Data Fig. 2a, b) and at the residues that support BRD4 binding (Extended Data Fig. 2c-f). This finding suggests that mTOR-mediated effect on BRD4-chromatin association and formation of the speckles may reflect changes in BRD4 or chromatin that preclude the BRD4 binding despite unaltered acetylated histone H4 levels.

In agreement with the negative impact of mTOR inhibition on BRD4 speckles, Torin1 treatment causes rapid and significant decline in BRD4 abundance at the promoters/ transcriptional start sites (TSS) of a large fraction (n = 2,386) of the protein coding genes expressed in the ES cells (n = 12,859) (Fig. 1c, Supplementary Table 1). The most pronounced decline of BRD4 binding is confined to Integrated Stress Response (ISR) genes that support cells response to different environmental stresses, including nutrient deprivation^12^ (Fig. 1d and Extended Data Fig. 1b). Many of the ISR genes that display a significant decline or even loss of BRD4, are controlled by the ATF4 transcription factor^13,14^, a key regulator of the stress response (Fig. 1d)^12,15,16^.

## Decline of BRD4 binding correlates with impaired transcription of BRD4-bound genes

The decline of BRD4 levels correlates with a rapid decline in gene transcription as judged by nascent gene transcriptional rate quantified by using metabolic incorporation of 4-thiouridine (4sU) in newly transcribed RNA (TT-seq)^17^ (Fig. 1e, left and middle panel and Extended Data Fig. 1c). Accordingly, changes in transcription precede a decline in the corresponding mRNA levels via total RNA sequencing (Fig. 1e, right panel and Extended Data Fig. 1d). Overall, a strong correlation between decline in BRD4 enrichment and transcriptional changes (Fig. 1f) suggests a possible causal role for impaired BRD4 binding in setting up a transcriptional program that supports cells response to mTOR inhibition that can lead to diapause. The ISR genes become activated at the early stages of nutrient deficiency and help cells to cope with the damaging effect of the stress^18–20^. It is well established that inhibition of mTOR following the initial response to nutrient deficiency helps to modulate the stress response levels by affecting translation of proteins that drive and support the stress^21,22^. Our observations show that mTOR suppression can mitigate stress response and hence promote cell survival not only via translational but also transcriptional mechanisms. The early timing of transcriptional response of ISR genes following mTOR inhibition suggests a direct link between mTOR signaling and transcription factors that drive ISR.

## Pharmacological inhibition of bromodomain-dependent BET-protein binding to chromatin triggers diapause-like state

The ability of mTOR to affect BRD4 suggested the possibility of circumventing diapause dependence on mTOR inhibition by inhibiting the BET proteins directly. The exposure of ES cells to incrementally increased concentration of I-BET151, the pharmacological inhibitor of the bromodomain-containing BET proteins^23^ results in the generation of ES cells, hereafter defined as I-BET resistant (I-BETR), that can exist in the presence of I-BET at a concentration prohibitive for the bromodomain-dependent chromatin binding (Fig. 2a, Extended Data Fig. 3a, b). The I-BETR ES cells maintain the expression of naïve ES cell marker alkaline phosphatase (AP), are smaller, and grow at a considerably slower rate than the control ES cells (Fig. 2a, b). The metabolic state of I-BETR ES cells is characterized by reduced levels of oxidative phosphorylation as judged by lower basal and maximal oxygen consumption rates (Fig. 2c, upper panel). The declined oxidative phosphorylation capacity is coupled with reduced levels of glycolysis as judged by reduced basal and maximal extracellular acidi cation rates before and after adding the mitochondrial electron transport chain inhibitors (Fig. 2c, lower panel). Finally, the I-BETR cells have decreased biosynthetic activity, as indicated by reduced overall and *de novo* RNA and protein synthesis (Fig. 2d, e). The described features of I-BETR ES cells are consistent with the previously described phenotypes of ES cells that were rendered diapause by mTOR inhibition or nutrient deprivation. Several lines of evidence suggest that acquiring the diapause-like state by I-BETR ES cells reflects cell adaptation rather than a selection of the rare ES cell variant. First, the progression towards the high concentration of I-BET was associated with minimal cell attrition at each step of increasing I-BET concentration (Extended Data Fig. 3a, b). Second, removal of I-BET reverses fully and quickly the diapause-like phenotype of I-BETR ES cells (Fig. 2b, c). Finally, after a short-term (12–14hours) removal of I-BET, the I-BETR ES cells could generate chimeras upon injection into the C57BL/6J mice-derived blastocysts (Fig. 2f). This finding underscores the unaltered pluripotent capacity of I-BETR ES cells and is consistent with the reversibility of diapause.

## Transcriptional reprogramming of I-BET resistant ES cells is consistent with diapause-like state

It is well established that chronic exposure to BET inhibitors in tumor cells can promote resistance to BET inhibitors^24,25^. This resistance is associated with adaptive changes in transcriptional control of the RNA Pol II transcribed genes in a fashion that reduce reliance of BET overall or bromodomain-dependent BET binding to chromatin^26^. Additionally, the BET inhibitor-resistant tumor cells undergo reprogramming of the cell kinome that adds to the complexity of adaptive processes that confer BET inhibitor resistance^27,28^. In the ES cells, the adaptive changes to BET inhibition append the transcriptional program characteristic for the control ES cells (Fig. 3a, Supplementary Table 2). At the height of these changes are cellular processes and biochemical pathways that have been previously linked to embryonic of ES cell diapause append^3,5^ (Fig. 3b, c, Supplementary Table 3, 4).

## mTOR inhibition leads to rapid transcriptional activation of negative regulators of MAP kinase

The broad changes in gene expression and metabolism in I-BETR cells have a negative impact on gene program that support ES cell differentiation into various lineages. The expression of differentiation-promoting genes in the I-BETR ES cells was as low as in ES cells treated with the mix of *GSK3β* and MAP kinase inhibitors (2i)^29^ (Fig. 3d). While some of the pluripotency supporting genes were down-regulated as well as in I-BETR ES cells, this effect was less uniform as compared the differentiation specific genes (Fig. 3d). The striking similarity between the impact of 2i and the long-lasting BET inhibition suggested a possible suppression of MAP kinase signaling during diapause. Indeed, a comparative analysis of gene expression patterns between I-BETR ES cells and previously described *Myc*-deficient ES cells revealed negative regulators of MAP kinase (MAPK) signaling pathway dual-specificity protein phosphatase *Dusp4*, *Dusp6*, and *Spry4* among commonly affected genes^30,31^. The transcriptional activation of negative regulators of MAP kinase signaling is among the earliest events that occur in response to mTOR inhibition in ES cells. Inhibition of mTOR upregulates genes encoding *Dusp1*, *Dusp4*, *Dusp6* as well as members of Sprouty family signaling antagonist *Spry1*, *Spry2 and Spry4* (Fig. 4a, Supplementary Table 5). Increased transcription of *Dusp4*, *Dusp6* and *Spry4* is accompanied by a rise of the corresponding protein levels and a concomitant decline of phosphorylated ERK (pERK) levels (Fig. 4b, upper panel, Extended Data Fig. 4a) following short-term inhibition of mTOR and maintained in the long-term mTOR inhibition induced diapause ES cells (Fig. 4b, lower panel, Extended Data Fig. 4b).

## Transcriptional activation of negative regulators of MAP kinase ensures the pluripotency during diapause

The transcriptional switch-off of MAP kinase occurs also in I-BETR ES cells, and *Dusp6, Dusp4* and *Spry4* are upregulated in I-BETR ES cells as compared to control ES cells (Fig. 4c, d). This transcriptional upregulation is associated with a profound decline in Erk activity (Fig. 4d, e). This shut-off the MAP kinase is critical for the I-BETR ES cell maintenance at the naïve state (Fig. 4f). The siRNA-mediated knockdown of *Dusp4, Dusp6*, or *Spry4* or the combined knockdown of all three genes led to the differentiation of I-BETR ES cells but did not substantially affect control ES cells, as measured by alkaline phosphatase expression and changes in ES cell morphology (Fig. 4f).

### mTOR inhibition rapidly reduces the levels of CIC transcriptional repressor at promoters of negative regulators of MAP kinase

What mechanism can contribute to the transcriptional activation of negative regulators of MAP kinase in response to mTOR or during the diapause? In ES cells or cells of other types, the negative regulators of MAP kinase are transcriptionally repressed by Capicua transcriptional repressor (CIC)^32^. The signal-induced activation of MAP kinase leads to dissociation of the Capicua transcriptional repressor (CIC) from promoters of genes encoding negative regulators of MAP kinase followed by these genes’ upregulation and inactivation of MAP kinase signaling^33^. This feedback mechanism ensures maintenance of MAP kinase signaling at physiological levels^33^. Our data show that the CIC is involved in the suppression of MAP kinase signaling in response to the diapause-induced signals. Pharmacological inhibition of mTOR in ES cells triggers a major decline in CIC occupancy at the promoters of genes encoding negative regulators of MAP kinase that coincides chronologically with the upregulation of these genes’ expression (Fig. 5a, b). The CIC was absent at the promoters of *Dusp4* and *Dusp6* genes in the diapause-like I-BETR ES cells (Fig. 5c). These data suggest a key role of mTOR-BRD4-CIC axis in maintenance of ES differentiation during diapause.

## Discussion

The described mTOR- and BET-controlled mechanism of diapause ES cell maintenance in an undifferentiated state has several implications. Our studies explain the high pluripotency potential of ES cells derived from the diapause embryos^34,35^. The metabolic dormancy is a hallmark of tissue resting stem cells or tumor stem cells^36–39^. It is possible that natural or artificial, i.e. drug-induced, suppression of BET binding to chromatin can play a defining role in setting up the transcriptional program responsible for the dormancy during stemness and *vice versa*. The metabolic dormancy or diapause-like state render cell resistance to adverse environmental conditions^40^. It is plausible that chronic I-BET treatment may endow some of the differentiated cells with enhanced resistance to toxic impacts. In agreement with this model, our unpublished data (SV, TZ, AT, AS) show a major protective effect of I-BET and associated metabolic dormancy on mouse neurons *in vitro* and *in vivo*. The induction of dormant or diapause-like state and concomitant support of stemness and protection against toxic impacts suggest a possible application of BET inhibitors for the purpose of tissue protection *in vitro*, e.g. organ preservation, or *in vivo* during conditions such as neurodegeneration or conditions that affect the stem cell pool. In this respect the protective effect of BET inhibition on type I diabetes in mice^41^ may reflect not only the suppression of T cell-driven inflammation but also direct enhancement of the pancreatic stem cell resistance to toxic impacts.

## Methods

### ES cell culture

The parental “CY2.4” ES line was derived from tyrosinase^−/−^ (white, albino) homozygous mouse embryos of B6(Cg)-Tyrc-2J/J C57BL/6J background^42^. ES cells were constantly maintained on the inactivated mouse embryonic fibroblasts (MEFs) by the treatment with 10 mg/ml final concentration of mitomycin C (M4287, Sigma-Aldrich) at 37°C with 5% CO_2_ for 2.5–3.0 hours. Parental ES cells were cultured in ES medium: 40% EmbryoMax DMEM (SLM-220, Millipore), 40% KnockOut DMEM (10829018, Thermo Fisher Scientific), 7.5% GemCell Fetal Bovine Serum (100–500, Gemini), 7.5% KnockOut Serum Replacement (10828028, Thermo Fisher Scientific), 0.1 mM EmbryoMax MEM non-essential amino acids (TMS-001-C, Millipore), 1 × EmbryoMax Nucleosides (ES-008-D, Millipore), 50 units/ml EmbryoMax penicillin/streptomycin (TMS-AB2-C, Millipore), 0.1 mM EmbryoMax 2-Mercaptoethanol (ES-007-E, Millipore), 2 mM L-Glutamine (TMS-002-C, Millipore) and 1,000 units/ml LIF (ESG1107, Millipore). 2i ES cells were cultured in 2i medium: 50% DMEM/F-12 (11320033, Thermo Fisher Scientific), 50% Neurobasal medium (21103049, Thermo Fisher Scientific), 1 × B27 supplements (17504044, Thermo Fisher Scientific), 1 × N2 supplements (17502048, Thermo Fisher Scientific), 0.1 mM EmbryoMax MEM non-essential amino acids (TMS-001-C, Millipore), 50 units/ml EmbryoMax penicillin/streptomycin (TMS-AB2-C, Millipore), 2 mM L-Glutamine (TMS-002-C, Millipore), 1% KnockOut Serum Replacement (10828028, Thermo Fisher Scientific), 10 mM 1-Thioglycerol (M6145, Sigma-Aldrich), 1 μM PD0325901 (72182, Stemcell Technologies), 3 μM CHIR99021 (72052, Stemcell Technologies), and 1,000 units/ml LIF (ESG1107, Millipore). I-BETR ES cells were cultured in ES medium containing 4 μM I-BET151 (GSK1210151A). Earle’s Balanced Salt Solution (EBSS) (24010043, Thermo Fisher Scientific) and leucine-free DMEM (226–024, Crystalgen) without serum were used for cell starvation assays. To inhibit the mTOR signaling, ES cells were maintained in ES medium containing 500nM Torin1 (S2827, Selleckchem).

### Generation of I-BET resistant (I-BETR) ES cells and chimerism

Parental CY2.4 albino B6 ES cells derived from B6(Cg)-Tyrc-2J/J embryos were constantly maintained on the mitomycin treated mouse embryonic fibroblasts (MEFs). Parental ES cells were rendered to I-BET resistant (I-BETR) with incrementally increase the concentration of I-BET151, starting from 250nM and ending with 4,000nM (4μM). The I-BETR ES cells were maintained one passage after removal of MEFs on the 0.5% gelatin treated plate before chimerism assay. After 12–14 hours of withdraw of I-BET from the culture medium (described before), the I-BETR ES cells were used for morula aggregations.

In brief, C57BL/6J inbred stock was used as embryo donors for aggregation with CY2.4 albino I-BETR ES cells and as *pseudo* pregnant surrogates. Embryos were collected at E2.5 from super-ovulated C57BL/6J females. Zona pellucida of embryos were removed by using acid Tyrode’s solution (T1788, Sigma). I-BETR ES cell colonies were treated with 0.05% Trypsin-EDTA for getting single cell suspension and 10–20 cells was aggregated with each zona-free embryo. Recombined chimeric embryos were cultured overnight in micro drops of EmbryoMax advanced KSOM embryo medium (MR-101-D, Sigma) covered with mineral oil (9305, Fujifilm) at 37 °C in 6% CO_2_. In the next morning, morulae and/or blastocysts were transferred into the uteri of *pseudo* pregnant E2.5 C57BL/6J surrogates. Chimerism was judged at birth by the presence of black eyes and later by the coat pigmentation.

### STED imaging of BRD4

ES cells were cultured and plated on Nunc Lab Tek II Chamber Slides (Thermo Scientific, 154534PK). Cells were fixed with 4% paraformaldehyde (Electron Microscopy Sciences, 15710) in DPBS for 15 minutes at RT and then washed twice with DPBS. Cells then were permeabilized with DPBS + 0.2% Triton (PBST) and blocked with 2% normal goat serum (Thermo Scientific, 50197Z) in PBST for 1 hour at RT. Cells were directly incubated with primary antibody BRD4 (1:500, Ab84776, Abcam) in 2% normal goat serum in PBST overnight at 4°C. Cells were washed twice with PBST and incubated with Alexa Fluor 488-conjugated goat anti-rabbit antibody (1:500, Thermo Scientific) containing 2% normal goat serum in PBST for 1 hour at RT. Cells were washed twice with PicoGreen (Invitrogen, P11496) containing DPBS and cover-slipped by using Prolong Gold antifade reagent (Invitrogen, P36930) and dried overnight. Imaging was performed by using the Zeiss LSM 780 Confocal Microscope (Zeiss, Oberkochen, DE). I-BET151 (10μM, 1 hour) and d-BET6 (100nM, 1 hour) were used as the positive control for the experiment.

### Analysis of STED imaging

Identification of the speckles: in brief, the individual .lif files were imported in the Cellprofiler pipeline, followed by splitting the images based on the different channels (STED for Brd4 staining and Picogreen for nuclear staining). The Picogreen image was used to identify the nuclei, inclusion criteria were set to be 450-pixel units diameter min. and 2000 max. Objects outside the range were discarded, and identified objects were labelled as “nuclei”. In a next step, a gaussian filter was applied on the STED image, and the earlier identified nuclei were used as object to mask the foci in the gaussian filtered STED image. Next, the foci within this mask were identified as speckles, and the following criteria were used: typical diameter of objects min. 6, max 24-pixel units; objects outside the diameter range or touching the border were discarded. The threshold strategy used was “global” and the thresholding method “otsu”, three class thresholding was applied, and pixels in middle intensity were assigned to the background. The threshold smoothing scale was 0, threshold correction factor 1.2 and lower and upper bounds on threshold were 0 and 1. No log transformation was included before thresholding, and the method to distinguish clumped objects and draw lines between clumped objects was based on intensity. The size of the smoothing filter for de-clumping was automatically calculated, and the value for local maxima that were closer than the minimum allowed distance was set to 4. The setting for fill holes in identified objects was set to “after both thresholding and de-clumping”. Additional steps were included to measure intensity, identify macromolecular speckles, measure object size, shape, filters to select based on intensity etc., but these have no effect on the identification of the speckles.

Determination of significant differences: the data were transferred from the excel file in a GraphPad Prism file, where the graphs were generated, and statistical analysis was performed. Determination of significant differences was done using an Ordinary One-Way ANOVA with Dunnett’s multiple comparison’s test, both for the Torin1 and controls (I-BET151 and d-BET6) graphs.

### Western blotting

1 × 10^6^ cells were collected and lysed on ice for 1 hour in 100 μl low salt (150mM NaCl) RIPA buffer (89900, Thermo Fisher Scientific) containing 2 × Protease Inhibitor Cocktail (P8340, Sigma-Aldrich), 1 × Phosphatase Inhibitor Cocktail Set I (524624, Millipore), 331nM TSA (1406, Tocris) and 2μl of Benzonase Endonuclease (70664, Millipore). 7.7 μl of 5M NaCl was add for every 100 μl of RIPA buffer to increase the salt concentration to 500mM. Cell lysate was then incubated on a spinning wheel in the cold room (4°C) for 1 hour, following with spinning down at 12,000g for 15min to separate the supernatant as the whole cell lysate. For western blotting, the whole cell lysate was mixed with 1 × NuPAGE LDS Sample Buffer (NP0007, Thermo Fisher Scientific) and 1 × NuPAGE Sample Reducing Agent (NP0004, Thermo Fisher Scientific) and denatured at 70°C for 10 minutes. 50,000 cells derived lysate was loaded into 12% Bis-Tris Protein gels (NP0341BOX, Thermo Fisher Scientific) and separated at 150 volts for 45–50 minutes. Protein gels then transferred onto Trans-Blot Turbo Mini 0.2 μm nitrocellulose membranes (1704158, Bio-Rad) and stained with ponceau S Staining Solution (A40000279, Thermo Fisher Scientific) to ensure equal transfer. Membranes were sequentially incubated with primary antibodies and secondary antibodies conjugated with horseradish peroxidase antibodies. After Incubation with SuperSignal West Dura Extended Duration Substrate (34075, Thermo Fisher Scientific), membranes were scanned by using ChemiDoc Imaging System (12003153, Bio-Rad) and the data was analyzed by Image Lab (Version 6.1). Western blotting for the investigation of pERK1/ERK2 and pERK1/ERK2 levels in the cells were performed by using stripping method. Membranes for pERK1/ERK2 detection was stripped with Restore PLUS Western Blot Stripping Buffer (46430, Thermo Fisher Scientific) for 15min at room temperature and blocked for 3 hours with 5% BSA TBST. Membranes were then reused for ERK1/ERK2 detection. Western blotting for the investigation of histone acetylation levels were also performed by using stripping method. In brief, membranes for detection of the histone acetylation modifications were stripped with Restore PLUS Western Blot Stripping Buffer (46430, Thermo Fisher Scientific) for 15min at room temperature and blocked for 3 hours with 5% BSA TBST. Membranes were then reused for histone H4 detection.

Antibodies used in the study including Anti-pERK1/ERK2 (9101S, Cell Signaling), Anti-ERK1/ERK2 (9102S, Cell Signaling), Anti-Dusp4 (ab216576, Abcam), Anti-Dusp6 (ab76310, Abcam), Anti-Spry4 (ab228712, Abcam), Rabbit monoclonal [EP1000Y] Anti-Histone H4 (acetyl K5) (ab51997, Abcam), Rabbit polyclonal anti-Histone H4K8ac antibody (61103, Active Motif), Rabbit monoclonal [EPR17906] to Histone H4 (acetyl K12) (ab177793, Abcam), Rabbit monoclonal [EPR1004] to Histone H4 (acetyl K16) (ab109463, Abcam), Rabbit monoclonal [EPR16606] to Histone H4 (acetyl K5 + K8 + K12 + K16) (ab177790, Abcam), Rabbit polyclonal to Histone H4 (ab10158, Abcam), Rabbit polyclonal anti-p70 S6 Kinase Antibody (9202S, Cell Signaling), Rabbit polyclonal anti-Phospho-p70 S6 Kinase (Thr389) Antibody (9205S, Cell Signaling), Rabbit polyclonal anti-Akt Antibody (9272S, Cell Signaling), Rabbit polyclonal anti-Akt Phospho-Akt (Ser473) Antibody (9271S, Cell Signaling), Anti-ACTIN (4970S, Cell Signaling). For HRP conjugated secondary antibodies used for western blotting, Goat polyclonal anti-Rabbit IgG (H+L) Secondary Antibody (31460, Thermo Fisher Scientific) and Goat polyclonal anti-Mouse IgG (H+L) Secondary Antibody (31430, Thermo Fisher Scientific) were applied in the study.

### Analysis of nascent transcription or *de-novo* protein synthesis

Total nascent transcription (5-Ethynyl Uridine, 5-EU) or *de novo* translation (O-propargyl-puromycin, OPP) were assessed in ES cells using Click-iT RNA Alexa Fluor 488 Imaging Kit and Click-iT Plus OPP Alexa Fluor 647 Kit respectively according to the manufacturer’s instructions (C10329 and C10458, Thermo Fisher Scientific). For analysis nascent RNA synthesis, ES cells were incubated for 1 hour in ES medium supplemented with 0.5 mM 5-EU. To measure *de novo* protein synthesis, ES cells were incubated for 30 minutes in ES medium supplemented with 10 μM OPP. Cells treated with 1mg/ml of Actinomycin D (15021, Cell Signaling) for 2 hours and 50μg/ml of Cycloheximide (239763, Millipore) for 4 hours were used as the negative controls for the assays. The collected cells were fixed for 15 min at room temperature in PBS supplemented with 3.7% paraformaldehyde (15710, Electron Microscopy Sciences) following by permeabilized in PBS supplemented with 1% BSA (A7906, Sigma-Aldrich) and 0.1% Saponin (SAE0073, Sigma-Aldrich) for 10 min at room temperature. The copper(I)-catalyzed alkyne-azide cycloaddition (CuAAC) was performed by using the indicated kits respectively. Samples were analyzed on the BD LSRII flow cytometry. Data were analyzed using the FlowJo software (Tree Star, Ashland, OR) and showed similar variance.

### Analysis of intracellular pERK1/2 level

1 × 10^6^ ES cells were collected and pelleted by spinning for 3min at 300g. Cells were resuspended in 1 ml 1 × DPBS (14190144, Thermo Fisher Scientific). Formaldehyde (28908, Thermo Fisher Scientific) was added to obtain a final concentration of 4% and the cells were fixed for 10 min at room temperature. Cells were then chilled on ice for 2min and washed twice with 1 ml 1× DPBS. Cells next were permeabilized by resuspending in 200 μl of 0.1% Saponin (SAE0073, Sigma-Aldrich) in 3% BSA DPBS and incubated for 15 min at room temperature. Permeabilization buffer was discarded, and cells were resuspended in 100 μl of 0.1% Saponin in 3% BSA DPBS containing 1:200 diluted Anti-pERK1/ERK2 (9101S, Cell Signaling), and incubated for 30 min at room temperature. Primary antibody was discarded, and cells were resuspended in 100 μl of 0.1% Saponin in 3% BSA DPBS containing 1:1,000 diluted Goat anti-Rabbit IgG (H+L) Cross-Adsorbed Secondary Antibody, Alexa Fluor 488 or Alexa Fluor 647 (Catalog # A-11008 or A-21244, Thermo Fisher Scientific) and incubated for 30 min at room temperature. Cells were washed twice by using 200 μl of 0.1% Saponin in 3% BSA DPBS following by one time washing with 1 × DPBS. Cells were eventually resuspended in 100 μl of 1× DPBS and analyzed on the BD LSRII flow cytometry. Data were analyzed using the FlowJo software (Tree Star, Ashland, OR) and showed similar variance.

### Quantitative RT-PCR (qRT-PCR)

Indicated ES cells were freshly collected, and total RNA was extracted by using Trizol reagent (15596026, Thermo Fisher Scientific) according to the manufacturer’s instructions. RNA was then treated with RNase free DNase set (79254, Qiagen) and cDNA was synthesized using reagents supplied with the first strand cDNA synthesis kit (11483188001, Roche). Quantitative real-time PCR was performed using SYBR Green I (04707516001, Roche) on a LightCycler 480 instrument (Roche). Primer sequences shown in Table S6.

### Alkaline phosphatase (AP) staining

Alkaline Phosphatase staining was performed using the Stemgent^®^ Alkaline Phosphatase Staining Kit II (Stemgent, 00–0055) according to the manufacturer’s instructions. In detail, 5 × 105 ES cells were seeded in a well of the 6-well plate a day before the experimental day. ES medium was aspirated, and cells were washed with 2 ml of 1 × PBST (1 × DPBS with 0.05% v/v of Tween20). Fix solution was added and incubated at room temperature for 2 minutes. Fix solution was then aspirated, and the fixed cells were washed with 2 ml of 1 × PBST. 1.5 ml of freshly prepared AP substrate solution was added in each well and incubated at room temperature for 12 minutes. Reaction was stopped by aspirating the AP Substrate Solution and the cells were washed twice with 2 ml of 1 × PBS. Cells were covered with 1 × PBS and directly used for imaging by using DMiL LED inverted tissue culture microscope (11521266, Leica) with Flexacam C1 camera. LAS X software was used for the data analysis.

### Seahorse metabolic assays

Control or I-BETR CY2.4 albino mouse embryonic stem (ES) cells were seeded in a Seahorse XF96 Cell Culture Microplate with the density of 5 × 10^4^ cells/well. Cells were cultured in medium with or without glucose overnight before the assay. For measuring OCR and ECAR rates in the I-BETR ES cells, medium without I-BET was applied to the I-BETR cells for indicated length of time. Culture media were exchanged to Agilent Seahorse XF Base Medium (102353–100, Agilent) 1 hour prior to the assay. OCR rates were measured by Agilent Seahorse XF Cell Mito Stress Test Kit (103015–100, Agilent) under basal conditions and after the sequential addition of 1 μM Oligomycin, 0.5 μM FCCP (carbonyl cyanide p-(trifluromethoxy) phenylhydrazone), 0.5 μM Rotenone and Antimycin A for the final concentrations according to the manufacturer’s instructions. ECAR rates were measured by Agilent Seahorse XF Glycolysis Stress Test Kit (103020–100, Agilent) under basal conditions and after the sequential addition of 0.5 μM Rotenone/Antimycin A and 50 mM 2-DG for the final concentrations according to the manufacturer’s instructions. Both the OCR and ECAR assays were performed by using Agilent Seahorse XFe96 Analyzer. All values were calculated per well and normalized to cell number for each experiment. Each experiment was normalized to the average of all controls across all the experiments performed (n=3). The metrics of OCR assay were calculated according to the following equations: Basal Respiration = and Maximum Respiration = . As proton efflux from live cells comprises both glycolytic and mitochondrial-derived acidification, for the ECAR assay, inhibition of mitochondrial function by Rotenone and Antimycin A enables calculation of mitochondrial-associated acidification. Subtraction of mitochondrial acidification to total proton efflux rate results in glycolytic proton efflux rate.

### RNAi-mediated knockdown of Dusp4, Dusp6 and Spry4

ES cells are trypsinized, counted and diluted in ES media without antibiotic to a density of 2.5 × 10^5^ cell/ml. 1 × 10^5^ ES cells were seeded in a 6-well plate containing feeders and incubated in at 37°C with 5% CO_2_ overnight. On the next day, mix 2.5 μl of 20 uM SMARTpool siRNA oligonucleotides (Dharmacon) targeted to mouse *Dusp4, Dusp6*, *Spry4* or a scrambled siRNA control (diluted in RNase-free buffer) with 197.5 μl of serum-free media in a microfuge tube (Tube 1) and incubate for 5 min at room temperature. In another tube, mix 4 μl of DharmaFECT^®^ 1 with 196 μl of serum-free media (Tube 2), mix and incubate for 5 min at room temperature. Transfer the contents of Tube 1 into Tube 2, mix gently, and incubate for another 20 min at room temperature. Remove ES media without antibiotic from the wells and add 400 μl of transfection medium to each well. After a 30 min incubation at room temperature, top up the volume to 2ml for each well by using ES media without antibiotic (~1.6ml). Mock-transfected cells were given DharmaFECT 1 transfection reagent only. Incubate the cells in a CO_2_ incubator for 48 hours and targeted gene expression was assessed by qRT-PCR. The order information for SMARTpool siRNA targeting of mouse *Dusp4, Dusp6* and *Spry4* are as follows: L-061306-00-0005, L-040050-00-0005 and L-059172-01-0005. The order information for mock non-targeting siRNA pool is D-001810-10.

### RNA-seq library preparation and analysis

#### Generation and sequencing of RNA-seq samples

Three or four replicates were used for samples in RNA-seq assays. Freshly collected cells were used for total RNA extraction by using TRIzol reagent (Thermo Fisher Scientific) according to the manufacturer’s instructions. Samples were either spiked-in or not with RNA Spike-In Mix (4456740, Thermo Fisher Scientific) following manufacturer’s recommendations. Ribosomal and mitochondrial RNA was removed, and library preparation were performed by using 250 ng total RNA in all samples based on TruSeq Stranded Total RNA Ribo-Zero Gold library prep kit for Illumina (RS-122–2301, Illumina). Samples were sequenced at Genomics Resource Center, The Rockefeller University on Illumina NextSeq500.

#### Generation of Transcript Annotations

The Get Gene Annotation (GGA) pipeline^43^ (https://github.com/AdelmanLab/GetGeneAnnotation_GGA; https://doi.org/10.5281/zenodo.5519927) was used to generate cell-type specific gene annotations using PRO-seq (control) and RNA-seq (+/− 1 μM Torin1) data generated in mESCs. Briefly 5’ end of PRO-seq reads were used to call transcription start sites (TSSs) and assign a dominant TSS for each gene. RNA-seq data was then quantified by kallisto (version 0.45.1)^44^ and used to assign a transcript end site (TES) location for each gene. These consensus gene annotations were used for all subsequent analyses.

#### RNA-seq analysis

Raw RNA-seq reads were quality filtered (mean quality score ≥20) and first mapped to ERCC spike sequences using STAR (version 2.7.3a)^45^. Reads not mapping to spike-in sequences were aligned to the mm10 genome using the following parameters: --quantMode TranscriptomeSAM GeneCounts --outSAMtype BAM SortedByCoordinate --loutMultimapperOrder Random --outSAMattrIHstart 0 --outFilterType BySJout --outFilterMismatchNmax 2 --alignSJoverhangMin 8 --outSAMstrandField intronMotif --outFilterIntronMotifs RemoveNoncanonicalUnannotated --alignIntronMin 20 --alignIntronMax 1000000 --alignMatesGapMax 1000000 --outWigType bedGraph --outWigNorm None --outFilterScoreMinOverLread 0 --outFilterMatchNminOverLread 0. Duplicates were also removed using STAR. Gene counts were generated using the featureCounts function of the Rsubread package (version 2.0.1)^46^ using the GGA annotations described above. Per-gene normalized counts values were then quantified using DESeq2 (version 1.26.0)^47^ and differentially expressed genes identified. Given comparable spike-recovery values between samples, normalization was performed using DESeq2 generated size factors.

#### Ontology analysis

For I-BETR RNA-seq comparisons, differentially expressed genes called by DESeq2 (Adjusted p-value < 0.001 and fold change > 2) were ranked by fold change. The top 225 upregulated genes were run through EnrichR^48^ (https://maayanlab.cloud/Enrichr/) and unique MSigDB Hallmark and Reactome categories passing significance thresholds (p-value < 0.001). The same process was repeated for the 200 most downregulated genes ranked by fold change.

### TT-seq library preparation and analysis

#### TT-seq library preparation

CY2.4 albino B6 ES cells were plated in T175 tissue culture flasks one day prior to treatment. On the day of treatment, Torin1 was added to a final concentration of 1 μM to ES media. For control treatment, an equal volume of vehicle was added to media for the same length of treatment. 500 mM 4sU (T4509, Millipore) was added to the samples exactly at 10min to the end of the given length of treatments. Cells were rinsed with room-temperature PBS and harvested using trypsin and quenched with cold DMEM + 10% FBS. After an additional wash with PBS, cells were counted and resuspended in 1 mL Trizol per 2 × 10^6^ cells. Samples were frozen immediately at −80°C until all the samples were ready for the downstream steps.

Prior to addition of chloroform to the lysates, samples were spiked-in with 5% cell counts based 4sU-labeled Drosophila S2 Trizol lysate (cells labeled with 4sU for 2 h and resuspended a concentration of 10 million cells/ml in Trizol). RNA was then isolated per the manufacturer’s protocol. The aqueous phase was precipitated by addition of 2.5 volumes of 100% ethanol with 1.4mM DTT, incubation at −20°C for 2h. Pellets were collected by centrifugation at 20,000g for 30 min at 4°C and washed twice with 500 mL of 75% ethanol before resuspension in 180 mL of nuclease free water. Aliquots were removed for quantification by spectrophotometry and analysis of RNA integrity by Agilent TapeStation 4200 using RNA high sensitivity tapes. Samples with RIN > 9.5 were used for further processing.

Total RNA from previous step was treated to remove residual DNA by using RNeasy Micro Kit (Qiagen, 74004) combined with Amplification Grade DNase I (18068015, Invitrogen) and Superase-In RNase inhibitor (AM2694, Thermo Fisher Scientific). In detail, 40 μl of 1M DTT was added into 1mL of RLT buffer prior to the procedures. 200 μl of RNA sample was mix with 700 μl of RLT buffer and 500 μl of 100% ethanol. Samples were loaded onto a MinElute column and spun for 1min at 3,500g. 700 μl of RW1 was then added and spun the column for 2min at 14,000g. 5 μl of DNase I and 35 μl of RDD mixture was then added onto the column and incubate at room temperature for 30 min. After incubation, 660μl of RW1 was added and column was spun for 1min at 14,000g. Wash the column with one time of 500 μl of RW1 buffer followed with one time of 500 μl of RPE buffer. Next, 500 μl of 80% ethanol was added and then spun the column for 2min at 14,000g. Repeat the last step once more and open the lid of the tubes for 5min at room temperature. 30 μl of RNase free water was added onto the column and incubate at room temperature for 2min and then spun at 200g for 2min. Add another 22 μl of RNase free water onto the column and repeat the same incubation following with spinning. Eventually the RNA samples were eluted by spun down for 2min at 14,000g and the concentration and quality of the samples were detected by Nanodrop and TapeStation respectively. Samples with RIN > 9.5 were used for further processing.

RNA samples were then chemically fragmented by addition of 20 mL cold 5 × fragmentation solution (375 mM Tris-HCl (pH 8.3), 562.5 mM KCl, 22.5 mM MgCl_2_) and incubation at 94°C for 3.0 minutes. At the end of the fragmentation time, RNA was placed immediately on ice and 25 mL of cold 250 mM EDTA was added. RNA was precipitated by addition of 1/10 volume of 5 M NaCl, 2.5 volumes of 100% ethanol and incubation at −20°C overnight. RNA samples were pelleted, washed, quantified, and analyzed again as described above. The TapeStation results should show bulk of the signal at 200–1,000nt with the peak size around 450–600nt.

Fragmented RNA was biotinylated as described in Duffy et al. (2015) with the following modifications: the biotinylation reaction was performed in a total volume of 200 μl and allowed to incubate for 45 min in the dark. Excess biotin was removed using chloroform:isoamyl alcohol and Phase-Lock-Gel (Heavy/High density) tube (Qiagen, 129056) were used to separate organic and aqueous phases. Biotinylated RNA was resuspended in 100 μl of nuclease-free water and aliquots taken to use as the total RNA input fraction. In parallel, Dynabeads MyOne Streptavidin C1 (Invitrogen, 65001) were prepared for binding to render them RNase-free: for each sample, 25 μl of beads were used and treated in batch to render them RNase free. The beads were incubated 10 min in the decon solution (100 mM NaOH, 50 mM NaCl) and placed on a magnetic stand. Then the beads were washed as follows: resuspending the beads fully for each wash, twice with 500 μl of 100 mM NaCl, twice with 500 μl of 1 × TTseq wash solution (100 mM Tris-HCl (pH 7.4), 10mM EDTA, 1 M NaCl, 0.05% Tween 20 in nuclease free water to which 1 μl SuperaseIN RNase Inhibitor (AM2694, Thermo Fisher Scientific) per 1mL solution is added prior to use), once in 500 μl of 0.3 × TTseq wash solution, and finally resuspended in 52 μl /sample of 0.3 × TTseq wash solution and 1 μl/sample Superase-In RNase inhibitor (Invitrogen, AM2696). Biotinylated RNA was heated at 65°C for 5min, placed on ice for 2 min, and mixed with 50 μl of prepared beads. Samples were rotated at room temperature in the dark for 30 min. After binding, the tubes were placed on a magnetic rack and the beads were washed 4 times with 500 μl of 1 × TT-seq wash solution to remove unbound RNA, fully resuspending for each wash. The wash solution was removed, and the beads resuspended in 50 μl of 100mM of DTT (freshly diluted from 1 M DTT stock) and incubated in the dark for 15 min at room temperature. The eluted RNA was recovered, and the elution step repeated with an additional 50 μl of 100mM of DTT.

The combined eluates were purified using Norgen RNA clean-up and concentration MicroElute kit (Norgen #61000) following the manufacturer’s instructions for small RNA enriched samples. Final elution was performed in 14 μl of nuclease-free water and the eluate was reapplied to the column for a total of two elution steps. A Qubit RNA high sensitivity reagent kit was used to quantify the input RNA and enriched RNA. Yields of 1–2% were typical. 340–400 ng of enriched RNA was used for library construction with the TruSeq RNA Library Preparation Kit v2, Set A and TruSeq RNA Library Preparation Kit v2, Set B (Illumina, RS-122–2001 and RS-122–2002) for individually indexed the samples. After 5 cycles of PCR, samples were removed from the thermal cycler and a test PCR was performed to determine the optimal number of final cycles. Libraries were pooled and pair-end sequenced on a NovaSeq SP: 2 × 50 flowcell.

#### TT-seq analysis

TT-seq reads were filtered (mean quality score ≥20) and mapped to the *Drosophila* (dm6) genome using STAR (version 2.7.3a). Reads not mapping to spike sequences were aligned to the mm10 using the following parameters: --quantMode TranscriptomeSAM GeneCounts --outSAMtype BAM SortedByCoordinate --loutMultimapperOrder Random --outSAMattrIHstart 0 --outFilterType BySJout --outFilterMismatchNmax 2 --alignSJoverhangMin 8 --outSAMstrandField intronMotif --outFilterIntronMotifs RemoveNoncanonicalUnannotated --alignIntronMin 20 --alignIntronMax 1000000 --alignMatesGapMax 1000000 --outWigType bedGraph --outWigNorm None --outFilterScoreMinOverLread 0 --outFilterMatchNminOverLread 0. Duplicates were also removed using STAR. Gene counts were generated using the featureCounts function of the Rsubread package (version 2.0.1) using the same GGA annotation used for RNA-seq. Differentially expressed genes and per-gene normalized counts values were then quantified using DESeq2 (version 1.26.0). Given comparable spike-recovery values between samples, normalization was performed using DESeq2 generated size factors. These normalization factors were also used to generate combined bedGraphs using coverage from all replicates across each condition.

#### Cluster analysis

For clustering of TT-seq data, protein-coding genes that were differentially expressed (Adjusted p-value < 0.005 and fold change > 1.5) at any time point of Torin-1 treatment were considered (1,065 genes). The clara function of the cluster package (R, version 2.1.4) was used to calculate dissimilarity between genes, resulting in 3 distinct clusters. Genes were represented in their respective clusters using the log_2_FoldChange values assigned via DESeq2.

#### Gene Set Enrichment Analysis

Gene set enrichment analysis (GSEA) was performed using normalized counts over exon-containing regions as calculated by DESeq2. Analysis was performed over the C5 GO Biological Processes gene set over 1000 permutations using the classic enrichment statistic setting.

### ChIP-seq library preparation and analysis

#### ChIP-seq library preparation

CY2.4 ES cells were plated at a density of 2.5 × 10^5^ cells/ml in ES medium (as described before) and cultured for 24 hours. (Cells were always grown on MEFs except one passage on gelatinized tissue culture plates before the experiment). ES cells were trypsinized and resuspended in a concentration of 1 × 10^6^ cells/ml in DPBS (room temperature). 2 × 10^7^ cells were used for each ChIP sample. Pierce 16% formaldehyde (28906, Thermo Fisher Scientific) was added to a final concentration of 1% and cross-linking was performed for 10 minutes at room temperature. Cross-linking was then terminated by adding 2.5M glycine to a final concentration of 0.125M at room temperature. Cells were washed with cold DPBS twice and pelleted by spun down at 1,260g for 6 minutes at 4°C. Cells were resuspended in LB1 buffer (50mM Hepes-KOH, pH7.9, 140mM NaCl, 1mM EDTA, 10% glycerol, 0.5% NP40, 1% Triton X-100, 1 × protease inhibitor, 1 × phosphatase inhibitor cocktail set I and 100μM TSA) and incubate for 20 minutes rotating at 4°C. Cells were pelleted for 6 minutes at 1,350 g at 4°C. Cells were then resuspended in LB2 buffer (10 mM Tris pH 8.0, 200 mM NaCl, 1 mM EDTA, 0.5 mM EGTA, 1 × protease inhibitor, 1 × phosphatase inhibitor cocktail set I and 100μM TSA) and incubated for 5 minutes rotating at 4°C. Cells were pelleted for 6 minutes at 1,350 g at 4°C and following by resuspended in LB3 (10 mM Tris pH 8.0, 100 mM NaCl, 1 mM EDTA, 0.5 mM EGTA, 0.1% sodium-deoxycholate, 0.5% sodium lauroyl sarcosinate, 1% Triton X-100, 1 × protease inhibitor, 1 × phosphatase inhibitor cocktail set I and 100μM TSA). Cells were sonicated using Focused-Ultra sonicator (E220, Covaris) for 12 minutes to generate chromatin fragments range from 200 to 600 bp of DNA in length according to the manufacturer’s instructions. Fragmented chromatin samples were then spun down at 20,000g for 30min at 4°C. Dynabeads anti-rabbit M-280 (11204D, Invitrogen) were pre-blocked with 0.5% BSA in 1 × DPBS and were incubated with 10 μg of BRD4 (Ab84776, Abcam), or 10 μg of Rabbit monoclonal [EPR16606] to Histone H4 (acetyl K5 + K8 + K12 + K16) (ab177790, Abcam), or 10 μg of CIC (PA1–46018, Thermo Fisher Scientific) antibodies for 8 hours. Dynabeads were washed twice by using 0.5% BSA in 1 × DPBS and fragmented chromatin was added to antibody-bead complex and incubated overnight by rotating at 4°C.

Beads were sequentially washed three times with wash buffer 1 (50mM Hepes pH7.5, 500mM NaCl, 1mM EDTA, 1mM EGTA, 1% Triton, 0.1% NaDoc, 0.1% SDS) and three times with wash buffer 2 (20mM Tris pH 8, 1mM EDTA, 250mM LiCl, 0.5% NP40, 0.5% NaDoc) at 4°C, followed by washing one time with 1 × TE buffer at room temperature. Pulled-down chromatin fragments was eluted by adding elution buffer (50 mM, Tris pH 8.0, 10 mM EDTA, 1% sodium dodecyl sulfate, 20ug/ml RNaseA) to the beads and incubated with shaking at 65°C for 30 minutes. Beads were then collected by spun down briefly and the supernatants were transferred into a new tube. Reversal of crosslinking was performed at 65°C overnight in a thermocycler. 2 μl of DNase-free RNase (11119915001, Roche) was added and incubated for 1 hour at 37°C following with 2 μl of Proteinase K treatment for 2 hours at 55°C. DNA was purified by using Qiagen PCR purification kit and resuspended in 10mM Tris-HCL.

The concentrations of the pulled-down DNA fragments were measured by Qubit dsDNA HS and BR Assay Kits (Q32851, Invitrogen) and ChIP libraries were prepared by using NEBNext Ultra II DNA Library Prep Kit for Illumina (E7103S, NEB) with size selection step. The concentration and size distribution of the libraries were then measured by Qubit dsDNA HS and BR Assay Kits and tapestation respectively. 100nM of each library was pooled together with a maximal 12 samples in a line for Illumina NextSeq500/NextSeq2000 sequencing in the Genomics Resource Center of the Rockefeller University.

#### ChIP-seq analysis

For ChIP-seq samples, raw reads were filtered (mean quality score ≥20) and adapter sequences were removed using cutadapt (version 1.14)^49^. Reads were then mapped to the mm10 reference genome using bowtie (version 1.2.2)^50^. Fragment midpoints were approximated by shifting coordinates downstream by 75bp and were converted to bedGraphs with a bin size of 50bp using the script bowtie2stdBedGraph.pl (https://github.com/AdelmanLab/NIH_scripts; https://doi.org/10.5281/zenodo.5519915). BedGraphs were then normalized by total sequencing depth and merged using custom scripts normalize_bedGraph.pl (https://doi.org/10.5281/zenodo.5519915) and bedgraphs2stdBedGraph.pl (https://doi.org/10.5281/zenodo.5519915) and converted to bigWigs for visualization on the UCSC genome browser.

Per-sample reads for +/− 1kb around global TSSs were determined using the custom script make_heatmap.pl (https://doi.org/10.5281/zenodo.5519915) on depth-normalized bedGraphs and summing reads in this window. Replicates were then compared to one another using a Pearson correlation coefficient to determine technical similarity.

#### ChIP-seq Metagenes and Heatmaps

Metagene plots of average read coverage were generated by summing depth-normalized reads in 50bp bins at each position for +/− 2kb around the dominant TSS of protein-coding genes as determined by GGA analysis using the custom script make_heatmap.pl (https://doi.org/10.5281/zenodo.5519915) and dividing by the number of sites. Heatmaps were generated using the Partek Genomics Suite (version 6.16.0812) using matrices of summed reads in 50bp bins for loci +/− 2kb around annotated TSSs.

#### Differential ChIP-seq binding at TSSs

For BRD4 ChIP-seq samples, total reads mapping to +/− 1kb around annotated TSSs of protein-coding genes per replicate were determined using a custom script extract_fragments.pl (https://doi.org/10.5281/zenodo.5519915). DESeq2 was used to compare 30 minutes Torin-1 treated samples to their corresponding control samples using the same depth-normalization factors determined for normalizing bedGraphs as custom size-factors. Differentially bound promoter regions were determined using an adjusted p-value threshold of < 0.05.

#### ChIP-seq quantitative PCR analysis

After antibody enrichment during chromatin immunoprecipitation (ChIP), purified DNA fragments by using Qiagen PCR purification kit was used as template for quantitative PCR (Q-PCR) analysis. In detail, 1/50 of input and 1/80 of ChIP samples were applied per PCR reaction. Quantitative real-time PCR was performed using SYBR Green I (04707516001, Roche) on a LightCycler 480 instrument (Roche). Dilution factor (66.7x) was considered during the analysis process. Primer sequences shown in Table S7.

### PRO-seq library preparation and analysis

#### PRO-seq library preparation

Control mESCs were permeabilized and subjected to PRO-seq library preparation as previously described^51^. Final libraries were pooled and sequenced paired end on an Illumina NovaSeq platform.

#### PRO-seq analyses

For mapping PRO-seq libraries, the following pipeline was used. Dual unique molecular identifiers (UMIs) 6nt in length were extracted using UMI tools (https://doi.org/10.1101/gr.209601.116). Read pairs were trimmed using cutadapt (version 1.14). An additional nucleotide was removed from read 1 (3’ end read) using seqtk (https://github.com/lh3/seqtk) to ensure non-overlapping read pairs in mapping. Reads were mapped to a combined genome comprised of both spike (dm6) and reference (mm10) genomes using bowtie2, which were then separated based upon respective genome. Using previously identified UMIs, mapped reads were deduplicated, then sorted using samtools (version 1.3.1)^52^. Reference reads were then separated into 5’ and 3’ end reads and subsequently converted to strand-specific bedGraph format using the script bowtie2stdBedGraph.pl (https://github.com/AdelmanLab/NIH_scripts; https://doi.org/10.5281/zenodo.5519915). Successfully mapped 5’ bedGraphs were used for determining gene annotation information.

### Accession numbers

The data discussed in this publication have been deposited in NCBI’s Gene Expression Omnibus (GEO) and are accessible through GEO Super Series accession number GSE249392.

### Quantification and statistical analysis

Data were processed using Prism 9 (GraphPad Software).

One-way ANOVA with Dunnett’s multiple comparisons test was used to determine the significant difference of BRD4-containing (BRD4+) speckles in the nucleus in control or Torin1-treated ES cells at indicated time points.

Mann-Whitney U test was used to determine the significant difference of BRD4 binding signals in control or Torin1-treated ES cells at indicated time points.

The Pearson correlation coefficient (R) values were used to determine the strength of the linear association between BRD4 and RNA signals.

For analysis of the correlation of gene expression between 160 ribosomal genes and all the expressed genes, the nonparametric Mann–Whitney U test with null hypothesis for randomly selected values X and Y from two populations was used. Statistical significance is indicated by *, p < 0.05; **, p < 0.01; ***, p < 0.001; and ****, p < 0.0001.

All the other assays were performed using two-tailed Student’s t tests (unless otherwise specified).

Statistical significance is indicated by n.s. no significance, *, p < 0.05; **, p < 0.01; ***, p < 0.001; and ****, p < 0.0001.

### Reporting summary

Further information on research design is available in the Nature Portfolio Reporting Summary linked to this article.

## Data Availability

All the nascent RNA-seq (TT-seq), bulk RNA-seq and ChIP-seq data have been deposited at GEO and are publicly available as of the date of publication. Accession numbers are listed in the key resources table (Super Series accession number GSE249392). Original western blot images are publicly available as of the date of publication. The DOI is listed in the key resources table. Microscopy data reported in this paper will be shared by the lead contact upon request. All original code is publicly available as of the date of publication. DOIs are listed in the key resources table. Any additional information required to reanalyze the data reported in this paper is available from the lead contact upon request. Unique and stable reagents generated in this study are available upon request.
